# Rapidly Progressive Probable Sporadic Creutzfeldt-Jakob Disease

**DOI:** 10.7759/cureus.23245

**Published:** 2022-03-17

**Authors:** Moustafa M Elziny, Shaimaa S Elsaid

**Affiliations:** 1 Department of Academic Internal Medicine and Geriatrics, University of Illinois at Chicago, Chicago, USA; 2 Department of Diagnostic and Interventional Radiology, National Cancer Institute, Cairo University, Cairo, EGY

**Keywords:** human prion disease, dementia, rapidly progressive, sporadic, creutzfeldt-jakob disease

## Abstract

Creutzfeldt-Jakob disease (CJD) is a rare, fatal brain infection caused by a human prion. Because CJD is associated with rapidly progressive neurological degeneration, it requires high suspicion for diagnosis. We report the case of a 79-year-old patient who presented with a rapidly progressive neurological clinical picture. The patient had positive 14-3-3 proteins in cerebrospinal fluid, electroencephalography was significant for periodic discharges, and magnetic resonance imaging of the brain showed both diffusion restriction and increased fluid-attenuated inversion recovery signal in different cortical regions, consistent with probable sporadic CJD infection. The patient was enrolled under hospice and palliative care. The patient passed away two months after the onset of her symptoms. We discuss the probable sporadic CJD diagnostic criteria and possible risk factors that might have led to a faster progressive course.

## Introduction

Creutzfeldt-Jakob disease (CJD) is a progressive neurodegenerative disease caused by abnormal infectious protein in the brain recognized as the prion. CJD is a rare disease with a worldwide reported incidence of one to two cases per million per year [1]. In a recent cohort study, the age-adjusted annual incidence of prion disease in the United States was estimated to be 1.5 cases per million from 2003 to 2015, and the median age of death was 67 years [2]. There are four main forms of the CJD. Sporadic CJD is the most common form, reported in 80-95% of CJD cases [3]. It is caused by the conformation of the non-pathogenic, cellular prion protein (PrP^C^) to the pathogenic scrapie-associated prion protein (PrP^Sc^), which occurs spontaneously or is mutation-induced [4]. The familial form is the second most common form, reported in 10-15% of the CJD cases. The familial form results from a pathogenic genetic mutation of the prion protein gene, which increases the conformation risk from PrP^C^ to PrP^Sc^ [3,4]. The iatrogenic CJD is reported in less than 1% of the CJD cases [3]; it is caused by PrP^Sc^ infection after exposure to contaminated human tissue from a patient with CJD [4]. Sporadic, familial, and iatrogenic CJD forms are sometimes called classic CJD. The variant CJD form is different from the classic forms of CJD; it is caused by meat ingestion from cows infected with bovine spongiform encephalopathy [4]. Variant CJD usually progresses slower than classic CJD, with initial psychiatric symptoms followed by neurologic symptoms. This report describes a patient with a rapidly progressive deteriorating course of a probable sporadic CJD leading to death in two months from the onset of symptoms.

## Case presentation

The patient was a 79-year-old Chinese female admitted to the University of Illinois hospital in August 2021 with a history of recent recurrent falls. The patient used to ambulate without assisted devices; she was feeling unsteady with difficulty in walking and mild lightheadedness, followed by recurrent falls. Despite using a Cantonese professional interpreter, the patient had slurred slow speech with limited verbal output. The patient’s limited answers affected her interview. Hence, most of the patient’s history was taken from her husband with the help of a Cantonese professional interpreter. She had a history of behavioral changes, suffered from personality changes, was acting differently, and had felt more anxious and more confused for six weeks. The patient also had a history of unintentional weight loss due to poor oral intake during this period. The patient’s husband also noticed a decrease in her short-term memory and a gradual decline in her living functional status. According to her husband, the patient was completely independent of activities of daily living and instrumental activities of daily living until July 2021.

The patient and her family denied any history of fever, significant vision changes, vertigo, presyncope, chest pain, shortness of breath, palpitations, urinary incontinence, motor weakness, numbness, delusions, or hallucination. They denied any recent travel history or exposure to sick contacts. Her medical history included a history of hypertension, type 2 diabetes mellitus, dyslipidemia, and gout. The patient was a non-smoker with a history of social alcohol drinking. She was not previously receiving any psychoactive or neurological medications. She had not had any previous surgeries or blood transfusions. She was not known to have any allergies. Her mother had a history of hypertension, and her eldest daughter was diagnosed with schizophrenia; however, there was no history of prion disease or dementia in her family.

The patient was vitally stable with no signs of autonomic dysfunction on admission. Her physical examination was significant for unsteady gait, difficulty speaking, left upper extremity jerking movements, and tremors. Her upper extremities reflexes were normal and symmetric; however, lower extremities reflexes were symmetrically brisk. The plantar reflex was absent. The patient’s cranial nerves examination, motor tone, sensation, and coordination were grossly normal. Montreal Cognitive Assessment (MoCA) test was difficult to be completed due to the patient’s cooperation.

On admission, complete blood count, liver function test, kidney function test, electrolytes, thyroid function test, partial thromboplastin time, prothrombin time, lactic acid, ammonia, carboxyhemoglobin, ceruloplasmin, vitamin B1, vitamin B12, and folate levels were all within normal range. Human immunodeficiency virus, heavy metal, and urine toxicology screenings were also normal.

Cerebrospinal fluid (CSF) studies were also performed. CSF specimen was markedly hypocellular with normal protein level and negative for neoplastic or other diagnostic cells. CSF glucose was mildly elevated (Table 1). CSF gram stain, CSF culture, acid-fast bacilli culture, venereal disease research laboratory (VDRL), herpes simplex virus-1 polymerase chain reaction (PCR), herpes simplex virus-2 PCR, varicella-zoster virus PCR, cytomegalovirus PCR, Epstein-Barr virus PCR, cryptococcal antigen, *Histoplasma* antigen, and *Blastomyces* antigen were all negative in CSF.

**Table 1:** Cerebrospinal fluid cell counts with differentials and chemistries. CSF = cerebrospinal fluid; WBC = white blood cell; RBC = red blood cell

CSF	Patient’s results	Reference range
Color	Colorless	
Clarity	Clear	Clear
WBC	1 WBC/UL	0–5 WBC/UL
RBC	0 RBC/UL	≤0 RBC/UL
Lymphocyte	90%	40–80%
Monocyte	10%	10–45%
Xanthochromia	Absent	Absent
Glucose	96 mg/dL	40–70 mg/dL
Protein	41 mg/dL	15–45 mg/dL

**Figure 1:**
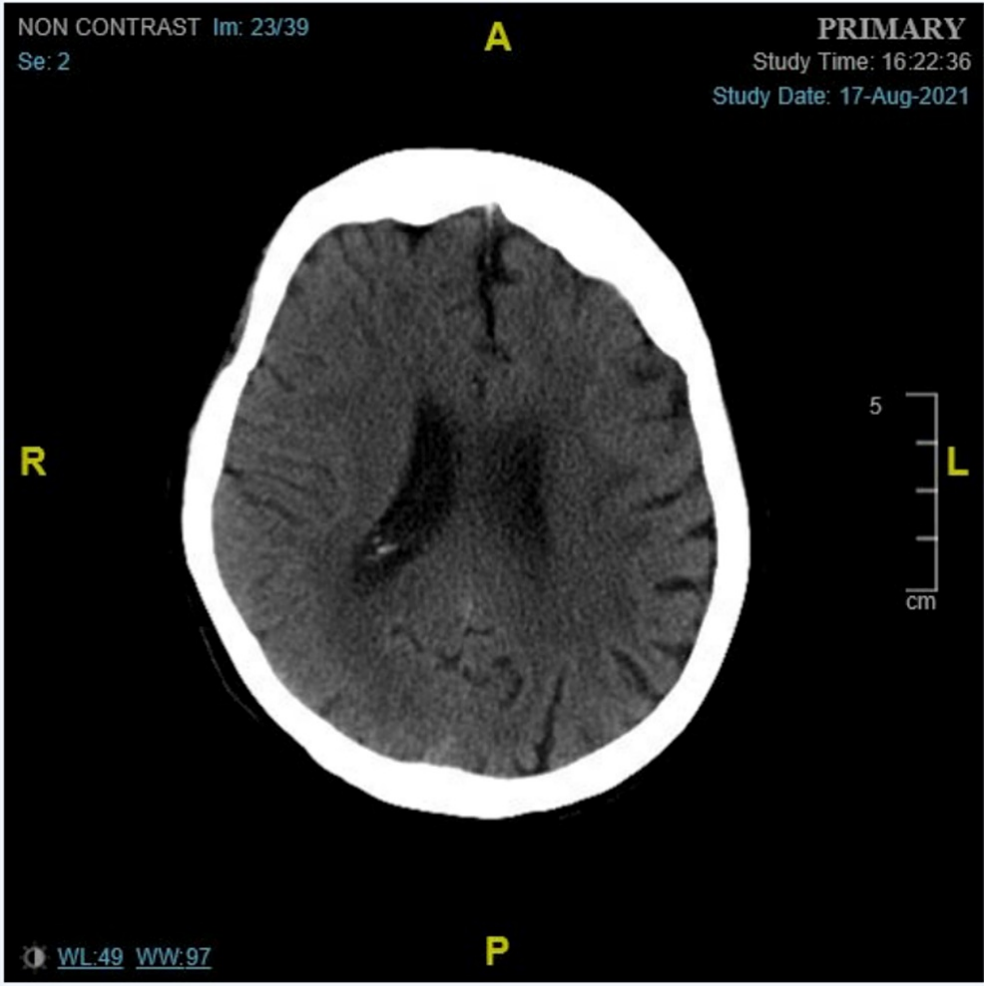
CT of the head showing areas of patchy low attenuation in the white matter of both cerebral hemispheres. CT: computed tomography

On admission, computed tomography (CT) of the head showed areas of patchy low attenuation in the white matter of both cerebral hemispheres are consistent with chronic small vessel ischemia. The ventricles were age-appropriate, with prominent cortical sulci consistent with the patient’s age. There were no signs of acute intracranial hemorrhage (Figure 1).

**Figure 2:**
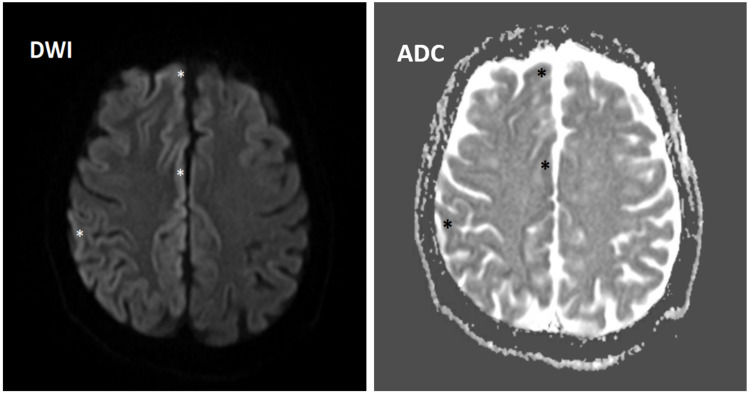
MRI of the brain showing diffusion restriction areas within the cortical sulci of the right frontal and parietal lobes (asterisks) in both DWI and ADC sequences. MRI = magnetic resonance imaging; DWI = diffusion-weighted images; ADC = apparent diffusion coefficient

**Figure 3:**
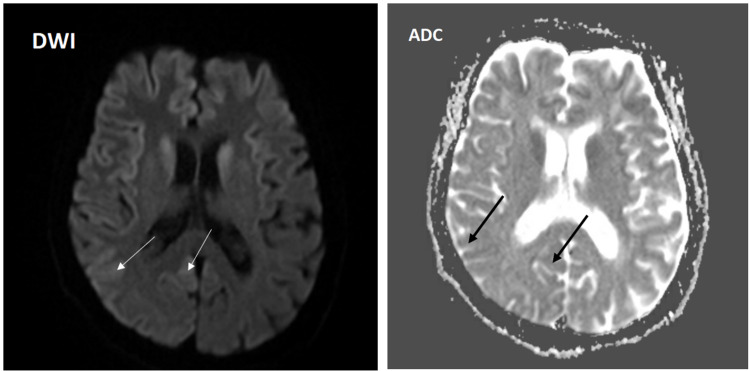
MRI of the brain showing diffusion restriction areas within the cortical sulci of the right temporal lobe posteriorly (arrows) in both DWI and ADC sequences. MRI = magnetic resonance imaging; DWI = diffusion-weighted images; ADC = apparent diffusion coefficient

**Figure 4:**
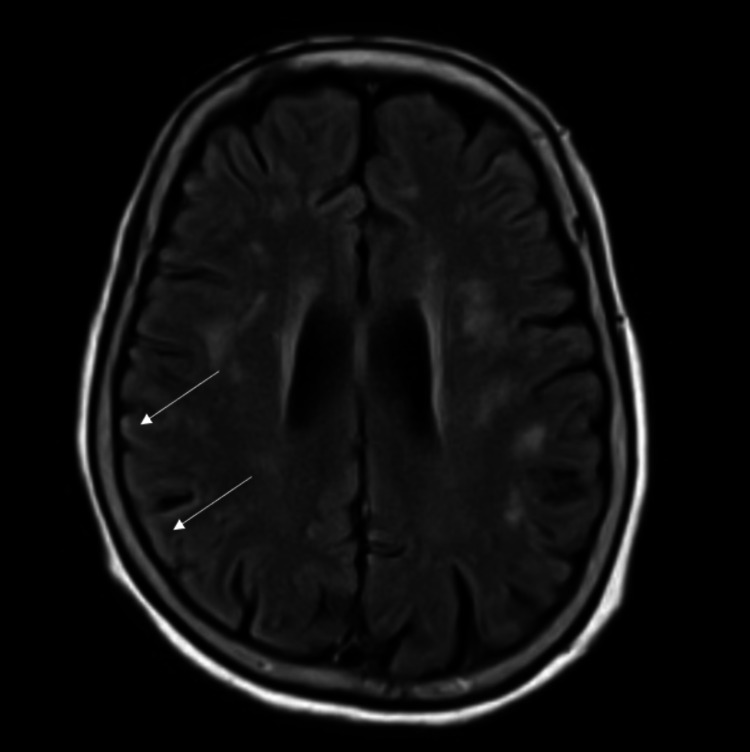
MRI brain scan showing increased FLAIR signal in the cortical sulci of the right parietal lobe (arrows). MRI = magnetic resonance imaging; FLAIR = fluid-attenuated inversion recovery

During hospitalization, magnetic resonance imaging (MRI) of the brain showed diffusion restriction areas within the cortical sulci of the right frontal and parietal lobes (Figure 2) and the right temporal lobe posteriorly (Figure 3). There was also an increased fluid-attenuated inversion recovery (FLAIR) signal in the cortical sulci of the right parietal lobe, likely consistent with CJD (Figure 4).

Continuous three-day electroencephalography (EEG) was significant for generalized periodic discharges and bifrontal high epileptogenic potentiality but no seizures.

During her hospital course, the patient’s mental status and orientation were rapidly deteriorating, and her responsiveness to questions and speech were decreasing. The patient was noted to have gradual difficulty swallowing, so she was placed on a puree diet. Moreover, she developed urinary retention, so a Foley catheter was placed. The patient had an uncomplicated *Escherichia coli* urinary tract infection which was treated and resolved with five days of intravenous ceftriaxone.

Several meetings and discussions were held with the family members, including her husband and her daughters. A considerable time was spent discussing her probable diagnosis of CJD, medical condition, and prognosis. Later, they agreed to enroll the patient under hospice and palliative care and transfer her to a skilled nursing facility. The patient’s condition continued to worsen progressively, and she died two months after the onset of her symptoms. Her CSF 14-3-3 proteins came positive later.

## Discussion

The Centers for Disease Control and Prevention (CDC) has established diagnostic criteria for CJD [5]. Given these criteria, our patient had rapidly progressive cognitive decline symptoms with a history of recent recurrent falls, upper extremity jerking movements, and tremors. She had positive 14-3-3 proteins in CSF, EEG was significant for periodic discharges, and MRI of the brain showed both diffusion restriction and increased FLAIR signal in different cortical regions. Based on the CDC criteria, our patient’s diagnosis is consistent with a probable sporadic CJD [5].

Our patient’s cognition and functionality rapidly declined, and she had behavioral changes, cerebellar signs, and myoclonus, which are common findings of sporadic CJD [6]. Additionally, she had no history of surgeries or blood transfusion with a negative family history of similar symptoms, ruling out the possibility of iatrogenic or familial forms.

Our patient had both diffusion restriction in different areas in the brain and increased FLAIR signal in the cortical sulci of the right parietal lobe. Abnormalities on FLAIR and diffusion-weighted images (DWI) are likely consistent with CJD. DWI abnormalities in MRI showed a high diagnostic value for CJD with 93.8% specificity and 92.3% sensitivity [7]. In a recent meta-analysis, MRI changes have 91% sensitivity and 97% specificity for CJD diagnosis [8]. Therefore, MRI is an important non-invasive diagnostic modality that should be performed on any CJD suspected patient to help in CJD diagnosis and exclude other differential diagnoses such as vascular, infectious, metabolic, malignant, and other causes of rapidly progressive cognitive decline.

CSF studies are another important diagnostic test to help with CJD diagnosis, and it is recommended to mainly rule out infectious causes of rapidly progressive cognitive decline. CSF 14-3-3 proteins are markers of neuronal destruction in the CSF; they can be detected in other diseases such as intracranial tumors and viral, metabolic, and Hashimoto’s encephalitis [9]. Therefore, CSF 14-3-3 proteins should be estimated to diagnose CJD in the appropriate clinical setting. In one study, the detection of 14-3-3 proteins in the CSF, as in our patient, associated with sensitivity of 94% and specificity of 84% correlated with the clinical diagnosis of CJD [10].

EEG findings in patients with CJD are variable and can range from non-specific diffuse slowing to the periodic sharp wave complexes (PSWC); seizures are uncommon findings in patients with sporadic CJD [11]. Our patient had a continuous three-day EEG which was significant for generalized periodic discharges. PSWC occurs in two-thirds of sporadic CJD patients with a positive predictive value of 95%, specificity of 91%, and sensitivity of 64% [11,12].

Various studies estimated that the patients with sporadic CJD have a median disease duration of five to six months [13-16]. Our patient had a total disease duration two months after the onset of her symptoms. In this case report, we tried to correlate the factors related to the rapidly progressive disease and decreased survival duration in our patient. The patients with older disease onset had a shorter duration of illness and more rapidly progressive disease [13,17]. Additionally, the less time between the disease onset and myoclonus observation was significantly associated with shorter disease duration [16]. Patients with positive 14-3-3 proteins were shown to be probably associated with rapid neuronal damage and shorter survival duration [13]. Further, patients with typical EEG changes have shorter survival than patients with non-specific EEG changes [13,16].

One of the limitations of our case report is that a brain biopsy was not done for the definitive diagnosis of sporadic CJD. On one hand, brain biopsy is considered the gold standard to diagnose CJD definitively. On the other hand, it is an expensive, invasive diagnostic tool with low sensitivity to confirm CJD diagnosis; in one study, brain biopsy only confirmed 11 (42%) out of 26 patients with autopsy-confirmed CJD [18]. Hence, brain biopsy is rarely indicated and should only be performed in the highly suspicious CJD cases when other non-invasive tests are non-conclusive [18].

## Conclusions

CJD is a rare progressive brain disease with no treatment that has proven to be effective or alter the course of the disease. CJD can cause rapidly progressive symptoms; however, it is essential to exclude other differential diagnoses such as central vascular accidents, infectious as viral encephalitis, metabolic, and malignant causes. Early suspicion and diagnosis should be made using the available diagnostic modalities such as MRI scan, EEG, and CSF analysis to prepare patients and their families for the CJD diagnosis, early goals of care discussion, and palliative and hospice care if desired.
